# The location of emotional intelligence measured by EQ-i in the personality and cognitive space: Are there gender differences?

**DOI:** 10.3389/fpsyg.2022.985847

**Published:** 2023-01-04

**Authors:** Marco Tommasi, Maria Rita Sergi, Laura Picconi, Aristide Saggino

**Affiliations:** Department of Medicine and Aging Sciences, University of Chieti-Pescara, Chieti, Italy

**Keywords:** trait emotional intelligence, gender differences, personality, fluid intelligence, ESEM

## Abstract

**Introduction:**

Emotional Intelligence (EI) is first described by Salovey and Mayer as the ability to perceive and understand emotions and the ability to use them as supports for thoughts. Despite the great notoriety of EI, its definition remains not completely clear. An operative definition of EI can be achieved by studying its connection with other individual characteristics such as gender, personality traits, and fluid intelligence.

**Methods:**

The sample was composed of 1,063 Italian subjects. A total of 330 participants were employed (31.0%; 57.9% men) and 702 were university students (66.0%; 38.7% men). The Emotional Quotient Inventory (EQ-i), one of the most used questionnaires in literature, was used to measure EI. The exploratory structural equation modeling (ESEM) was used to assess the role of personality traits (five-factor model of personality) and fluid intelligence in EI. Statistical analyses on differences between men and women means of total and subscale EQ-i scores were estimated to evaluate whether EI, measured by EQ-i, is influenced by gender. Furthermore, a Multigroup Confirmatory Factor Analysis was conducted to assess measurement invariance in relation to gender groups.

**Results:**

Emotional Intelligence, measured by EQ-i, is prevalently connected with personality traits rather than fluid intelligence. Furthermore, men outperformed women in the Intrapersonal and Stress Management EI factors, and women outperformed men in the Interpersonal EI factor. No difference in the means of the EI total score and EI latent general factor did not differ between gender groups.

**Conclusion:**

Emotional Intelligence, measured by EQ-i, can be conceptually considered as a Trait EI. Furthermore, men are more capable to cope with negative events and to control impulses, while women are more able to distinguish, recognize, and comprehend others’ emotions.

## 1. Introduction

During a survey, Sternberg (1985) asked common people to give a definition of intelligence. Beyond the ability of problem-solving and reasoning, he found that another ability was also considered important: the ability to accept other people, to comprehend their needs, and the sensibility to their emotional states. The idea that intelligence should also include social and empathic abilities, which was not new in psychology and was defined as *Social Intelligence* (Thorndike, 1920; Walker and Foley, 1973). Social Intelligence was not a well-defined concept (Cronbach, 1960), but it received strong attention from researchers studying individual differences (Sternberg et al., 1981).

Subsequently, Salovey and Mayer (1990) introduced the term *Emotional Intelligence* (EI) as a substitution for social intelligence. According to these authors, EI consists of three types of abilities: The ability to appraise one’s own and others’ emotions; the ability to regulate and express emotions; and the ability to use information about one’s own and others’ emotions to improve personal thinking and actions. The concept of EI became a famous and important topic in psychological research in 1998 (Hughes and Evans, 2018), especially after the publication of Goleman’s book: “Emotional Intelligence: Why it can matter more than IQ” (Goleman, 1995).

One of the most important problems with the concept of EI was operationalizing it to develop valid measuring instruments. In other words, a modulization of EI was necessary to obtain valid and consistent measures of EI. Different models of EI were proposed in literature. In the Mayer and Salovey model (Mayer and Salovey, 1997), it defined EI as a form of *intelligence* or *mental ability* (*ability EI*) in its traditional meaning-ability in learning, in problem-solving, in dealing new situations, and in achieving lifegoals (Legg and Hutter, 2007). Petrides and Furnham (2000) proposed a different model of EI, *Trait EI* or *Trait Emotional Self-Efficacy Trait EI.* It was defined as a set of emotional perceptions measured *via* questionnaires and rating scales (Petrides et al., 2007) and was a type of personality factor, separated from intelligence (Petrides, 2010), even if emotion recognition and regulation are considered important components of EI (Petrides and Furnham, 2000).

Other EI models are defined as mixed models, in which the EI is not specifically considered as an ability or a trait but more as a combination of them (Ackley, 2016; Dhani and Sharma, 2016; Hughes and Evans, 2018; O’Connor et al., 2019; Bru-Luna et al., 2021; Robles-Bello et al., 2021). Among the mixed models of EI, the Bar-On model of EI (Bar-On, 1997, 2000, 2006) considered it as a mixture of social and emotional skills, consisting of people’s ability to understand their and others’ emotions and to express them. The model consisted of five EI factors: The *Intrapersonal Emotional Intelligence* (Intrapersonal EI), which is self-awareness, the knowledge of one’s own skills to achieve goals, and the ability to understand and express one’s emotions and thoughts appropriately; the *Interpersonal Emotional Intelligence* (Interpersonal EI), which is the ability to understand others’ emotions; the *Stress Management Emotional Intelligence* (Stress Management EI), which is related to the ability to face stressful situations and to control impulses; the *Adaptability Emotional Intelligence* (Adaptability EI), which is the ability to adapt our own emotions to the social context; the *General Mood Emotional Intelligence* (General Mood EI), which is related to the satisfaction with life and optimism. These data show that EI is summarized in three models: Ability, trait, and mixed. All these models influence the construction of instruments of measure of EI. Indeed, recent literature points to the following problems that must be solved to obtain valid measures of EI: Coherence of the EI meaning for all researchers; validity and reliability of EI measures; impact of gender and age on EI; EI role in getting a successful life; predictive EI validity of human behavior; presence of neural and genetic correlates of EI (Zeidner et al., 2008; Di Fabio, 2010; Petrides, 2010; Saggino et al., 2013; O’Connor et al., 2019; Picconi et al., 2019; Allahyari, 2020; Bru-Luna et al., 2021; Sergi et al., 2021).

### 1.1. Relation between personality traits and self-report assessment tools of EI

In a recent meta-analysis, 95 studies (*N* = 30.198) were analyzed to verify the association between EI self-report instruments and personality factors (Van der Linden et al., 2017). The meta-analysis showed significant correlations between Trait EI measured through the TEIQue and the Wong and Law Emotional Intelligence (WLEIS) and all personality factors. Correlations ranged from *r* = 0.38 for Openness to *r* = −0.68 for Neuroticism. These results were confirmed by another research, in which the dimensions of TEIQue had significant connections with Neuroticism and Extraversion (Pérez-González and Sanchez-Ruiz, 2014). Petrides et al. (2010) found positive and significant correlations between Trait EI (measured through the TEIQue) and Extraversion, Openness, Agreeableness, and Conscientiousness (correlations ranged from *r* = 0.24 to *r* = 0.54). Abe et al. (2018) found a negative correlation between Neuroticism and the TEIQue. Another study found positive and significant correlations between personality and the TEIQue, ranging in absolute values from *r* = 0.61 to *r* = 0.78 in a sample of university students (Van der Linden et al., 2012). Petrides et al. (2007) found that Trait EI is a separate factor from the personality factors, but strictly connected with them. Other research confirmed the connection between the Trait EI and the five-factors of personality (Petrides et al., 2010; Alghamdi et al., 2017; Musek, 2017; Abe et al., 2018). These data are in line with research that found high correlations between big five traits and Trait EI (*r* > 0.85), while the Ability EI had a moderate correlation with the personality (from *r* = 0.20 to *r* = 0.30) (Van der Linden et al., 2016). In literature, there are few studies that examine this latter association, due to the scoring problem and the small area of the research about the instruments of the Ability of EI. Indeed, a recent meta-analysis showed as the Ability EI accounted for 28% of the shared variance with the personality; while the Trait EI accounted for 41% of the shared variance with the personality (Van der Linden et al., 2017).

### 1.2. Relation intelligence and self-report assessment tools of EI

In relation to the cognitive aspect of EI, there are no clear indications of the connection between EI self-report instruments and cognitive abilities (Pardeller et al., 2017). Some studies did not evidence a strong connection between EI self-report instruments and cognitive abilities measured through Raven’s Progressive Matrices (Raven et al., 1977; Bastian et al., 2005). This result was not confirmed by Udayar et al. (2018), who found significant correlations between TEIQue and the Raven’s (1938) Standard Progressive matrices (*r* = 0.14). A recent study (Nath et al., 2015) found a negative correlation between the EQ Test Questionnaire (Singh, 2006) and the Wechsler’s Adult Intelligence Test. Halimi et al. (2020) showed that Trait EI, measured *via* self-report instruments, predicted academic success. In any case, cognitive ability (mathematical and linguistic performance) was a moderator in association between the Trait EI and academic success and an incremental role of the Trait EI on fluid intelligence (Petrides et al., 2018). In particular, fluid intelligence (gf) is derived from Cattell’s theory of intelligence (Schneider and McGrew, 2012). In this theory, general intelligence (g) is split into crystallized intelligence (gc) and fluid intelligence. Gc is the ability to solve problems based on acquired experience, whereas the gf is the ability to solve problems without specific acquired experience (Deary et al., 2007). Both factors of general intelligence are associated with life outcomes, such as mortality, personality, and emotional aspects (Simpson-Kent et al., 2020). For example, high emotional stability is associated with higher levels of cognitive tasks, as well as extraversion is associated with attention (Nechtelberger et al., 2020). In particular, the gf factor is based on rational processes, associated with the general factor g, which remains stable and is predictive of several outcomes during life (Van der Linden et al., 2017). Furthermore, recent studies show the association between EI (measured through the EQ-i) and the Functional Connectivity in the Superior Parietal Lobule (Li et al., 2020a). In particular, higher Emotional Intelligence correlates with the “Default Mode Network” (DMN), which includes the medial-frontal cortex and parietal areas. A lower EI correlates with the “Dorsal and Anterior Network” (DAN) (Ling et al., 2019). The DMN is associated with the “rest” phase when an individual is not engaged in tasks with any attentional or cognitive goal; the DAN network plays a crucial role in attentional tasks and provides spatial coding (Vossel et al., 2014; Mak et al., 2017). The DMN has a key role in social cognition (Schilbach et al., 2008; Mars et al., 2012; Dodell-Feder et al., 2014). In particular, the medial-frontal cortex is associated with self and other representations. Indeed, a lesion in this area is linked to a deficit in social relationships, such as poor empathy, thinking about the future, and the ability to attribute mental states to others. The latter are important aspects of EI (Schilbach et al., 2006). These data confirmed the unclear construct validity of the self-report EI instruments (Pardeller et al., 2017).

### 1.3. Gender differences in the self-report assessment tools of EI

Some authors found no significant differences between men and women in EI (Petrides and Furnham, 2000; Fernandez-Berrocal et al., 2004; Poulou, 2010; Pérez-Díaz and Petrides, 2021), while other studies showed that women have higher levels of EI than men (Ciarrochi et al., 2001; Schutte et al., 2002; Katyal and Awasthi, 2005; Van Rooy et al., 2005; Craig et al., 2009; Whitman et al., 2009; Petrides, 2021). A recent study showed that men have higher levels of EI than women (Perazzo et al., 2021).

Research has shown that men and women can have different performances in every single subscale of self-report EI instruments: In some subscales, men outperform women, and in others, women outperform men (Petrides and Furnham, 2000; Khalili, 2011; Toyota, 2011; Aiyppa and Balakrishna Acharya, 2014). Gomez-Baya et al. (2017) studied the perceived EI using the Trait Meta Mood Scale in a sample of adolescents (TMMS; Fernandez-Berrocal et al., 2004) and showed that girls have higher attention to emotions. Malinauskas et al. (2018) studied gender differences in EI using the Emotional Intelligence Scale (EIS; Schutte et al., 1998). They found that women scored higher in all domains than men.

All studies on gender differences in EI compared the means of raw scores obtained by men and women in self-report instruments to compute statistical significance. Few studies examined the measurement invariance of self-report measures. The measurement invariance of the EI factorial models was mainly estimated to assess the cross-cultural validity of EI measures (Ekermans et al., 2011; Li et al., 2012). Few studies examined the measurement invariance of EI measures in men and women. Whitman et al. (2009) explored the structural equivalence between men and women of Wong and Law Emotional Intelligence. Tsaousis and Kazi (2013) analyzed the measurement invariance between genders of the Greek Scale of Emotional Intelligence. The former study showed that, in the *Use of Emotions* factor, women scored higher than men. The latter study showed that the scale was, substantially, equivalent both for men and women, even if women obtained higher latent means in the *Expression and Recognition* and *Caring and Empathy* factors. Pérez-Díaz et al. (2021) found scalar invariance (equal latent means) between genders in a sample of healthy subjects measured through the TEIQue. Furthermore, Pérez-Díaz et al. (2022) found a metric (equal factor loading) and scalar (equal latent means) invariance in a clinical sample between men and women of the TEIQue.

### 1.4. Relation between personality traits, cognitive abilities, and EQ-i scores

Since the EQ-i is a questionnaire based on a conception of EI as composed of mixed factors of cognitive intelligence and personality, the measures obtained with the EQ-i should relate to both personality and intelligence.

Bar-On (1997) compared the measures of the EQ-i facet scales with those obtained with Cattell’s 16-PF and the Eysenck Personality Questionnaire (EPQ). He found that many of the EQ-i facet scales correlated significantly with the Emotional Stability, Social Boldness, Apprehension, Perfectionism, and Tension factor of the 16-PF and correlated positively with the Extraversion factor and negatively with the Neuroticism factor of the EPQ (Bar-On, 1997). Dawda and Hart (2000) found significant correlations between the five primary factors of EQ-i and the five personality factors, except for the Openness factor. The authors also found gender differences in the correlations between EI and personality. The highest correlations were found between the Mood factor of the EQ-i and Neuroticism in men and women. Van der Zee et al. (2002) correlated the scores of the EQ-i five scales with those of the five scales of the Connector-P, a questionnaire developed to measure the five personality factors in work contexts. They found that the five-factors of personality significantly predicted the dimensions of Empathy, Autonomy, and Emotional Control of the EQ-i. However, they found both positive and negative correlations between EI and personality traits. Franco and Tappatà (2009) found that all the EQ-i five dimensions were positively correlated with the five traits of personality measured by the Big Five Observer. These strong associations were confirmed by Van der Linden et al. (2017), who found an overlap between EI and the general factor of personality. These results were not in line with Davis and Wigelsworth (2018), who did not find significant correlations between Neuroticism and Interpersonal factor. The highest association was between Neuroticism and Stress dimension (*r* = 0.48; *p* < 0.01). These data show that there are incongruences in correlations between the EQ-i scales and personality factors, especially the big five-factors.

No data are available in the current literature on the relationship between EQ-i scores and measures of intelligence, even if most studies analyze associations between EI and gf. In particular, fluid intelligence measures abstract reasoning and the ability to organize a complex stimulus. Furthermore, gf is not a specific ability, such as memory and language (Downey et al., 2014; Romanelli and Saggino, 2014; Li et al., 2020a). Finally, the gf is associated with behavioral regulation, through cognitive resources, when an emotional conflict exists (Li et al., 2020b).

### 1.5. Gender invariance in the EQ-i

Research results on gender effects on EQ-i scores are ambiguous. Reiff et al. (2001) showed that the Interpersonal EI factor was higher in women than in men. Furthermore, De Weerdt and Rossi (2012) found gender differences in a sample of 967 participants. The *t*-tests showed that men obtained higher means in the Intrapersonal, Stress Management, and General Mood factor. Women obtained higher means in the Interpersonal factor. This finding was confirmed by Bar-On et al. (2000) and Alumran and Punamäki (2008). The Italian standardization of the EQ-i was conducted on a sample of 1,353 subjects (49.5% men) with a mean age of 41.52 years (*SD* = 15.88) (Franco and Tappatà, 2009). The authors found that men scored higher than women in the Stress Management and General Mood factor, while women scored higher in the Interpersonal EI factor.

Some studies found no gender differences in the global score of EQ-i (Palmer et al., 2003; Saklofske et al., 2007), while other studies reported significant gender differences in the global EQ-i score (Ahmad et al., 2009). Davis and Wigelsworth (2018) found higher scores in women in the Interpersonal, Adaptability, and EQ-i global score in a sample of adolescents. Paskaran and Azman (2020) found higher scores in men in all domains, while they found no differences in EQ-I global scores.

Finally, several studies show that women have higher EI scores than men, regardless of the different instruments used. This can be explained in different ways of processing emotional stimuli. Indeed, women are more accurate in this process through better facial recognition of emotions (Batool and Khalid, 2009; Śmieja et al., 2014; Bru-Luna et al., 2021).

It must be mentioned that no author performed in these studies a confirmatory multigroup factor analysis to assess the measurement invariance of the EQ-i questionnaire.

### 1.6. Other adaptations of the EQ-i

The Emotional Intelligence Questionnaire-Youth Version (EQ-i: YV; Bar-On and Parker, 2000) consisted of 60 items to measure EI in children and adolescents. Responses to each item are given using a 4-point scale from 1 (“very seldom true or not true for me”) to 4 (“very often true of me or true of me”). In the Spanish version, the instrument included five principal dimensions: Intrapersonal EI, Interpersonal EI, Adaptability EI, Stress Management EI, and General Mood EI. The instrument had good reliability (α = 0.89). The factorial structure was not confirmed by Pérez Fuentes et al. (2014), who proposed a new adaptation of the EQ-i: YV in a sample of Spanish older university students. The instrument consisted of 20 items with a five-factor structure, and it was called Brief Emotional Intelligence Inventory for Senior Citizens (EQ-I-M20) (Cronbach’s Alpha ranged from α = 0.57 for the Intrapersonal factor to α = 0.83 for the General Mood factor). Italian validation of the EQ-i: YV (Sannio Fancello and Cianchetti, 2012) included 60 items, with seven dimensions: Intrapersonal EI, Interpersonal EI, Adaptability EI, Stress Management EI, General Mood EI, Total EI, and Positive Impression. Cronbach’s Alpha ranged from α = 0.62 for the Intrapersonal factor to α = 0.88 for the General Mood EI in the men’s sample, while internal consistency ranged from α = 0.61 for the Intrapersonal dimension to α = 0.87 for the General Mood EI in the women’s sample.

The Emotional Quotient Inventory 2.0 (EQ-I 2.0; Multi-Health System, 2011) was the revised version of the EQ-i. The EQ-i 2.0 measured the following EI dimensions (Self-Perception, Self-Expression, Interpersonal, Decision Making, and Stress Management) and 15 subscales: Self-regard, Self-actualization, Emotional Self-awareness, Emotional Expression, Assertiveness, Independence, Interpersonal, Empathy, Social Responsibility, Problem-Solving, Reality Testing, Impulse Control, Flexibility, Stress Tolerance, and Optimism. The factor structure showed poor fit indices for Optimism, Impulse Control, and Empathy (Root Mean Square Error of Approximation; RMSEA = 0.192, RMSEA = 0.149, RMSEA = 0.155, respectively). The reliability ranged from ω = 0.72 for Flexibility to ω = 0.91 for Self-Perception, Interpersonal, Decision Making, and Stress Management (Van Zyl, 2014). The EQ-i 2.0 measured the EI associated with leadership and job performance (from *r* = 0.20 to *r* = 0.47) (Stein and Deonarine, 2015; Ackley, 2016).

The analyzed paragraphs show that the EI has several psychometric problems, including (1) What does this construct measure? EI definitions are multiple and unclear. Therefore, it is essential to define the construct in a clear and shared way; (2) Which instruments measure the shared construct of EI? and (3) Is the EI an autonomous construct or a mixture of personality factors?

### 1.7. Aims of the study

Due to contradictory data in the literature, the first aim of our study was to analyze the association among self-report instruments of EI (measured with the EQ-i), personality factors, and intelligence–in particular fluid intelligence–to clarify whether the EQ-i could be considered as a measure of Trait EI or ability EI or both. We hypothesized several associations between the EI factor, measured by the EQ-i, and variables related to personality factors or intelligence. In particular, the first hypothesis was divided into three sub-hypotheses:

1. H1a: EI factor (measured with the EQ-i) has significant loadings only with personality factors.
2. H1b: EI factor (measured with the EQ-i) has significant loadings with fluid intelligence.
3. H1c: EI (measured with the EQ-i) has significant loadings both with personality and fluid intelligence.

The second aim of this study was to investigate the impact of gender differences on EQ-i scores. We hypothesized that EQ-i scores were influenced by gender characteristics at a global level or in specific EI domains. The second hypothesis was divided into three sub-hypotheses:

1. H2a: Gender influences the global score of EQ-i, but not the score of its specific subscales.
2. H2b: Gender influences the score of the EQ-i-specific subscales, but not its global score.
3. H2c: Gender influences both the global and specific subscales score of EQ-i.

## 2. Materials and methods

*Participants.* A total of 1,063 subjects composed the sample; 578 participants were women (54.4%) and 485 were men (45.6%), with a mean of 26.65 years and an SD of 11.92 years. In all, 330 participants were employed (31.0%; 57.9% men); 702 were university students (66.0%; 38.7% men); missing = 2.9%.

### 2.1. Materials

*Measures of EI*. EI was measured with the EQ-*i*, a 133-item self-report inventory based on Bar-On’s model of EI (Bar-On, 1997, 2004). Responses to each item are given using a 5-point scale from 1 (“very seldom true or not true for me”) to 5 (“very often true of me or true of me”). The EQ-i includes a global score and scores of each of the five principal dimensions: Intrapersonal EI (“*It is quite for me to express my feeling*,” “*I am aware of my emotions*,” “*I feel safe in most situations*”), Interpersonal EI (“*I am unable to show affection*,” “*I like helping people*,” “*I am unable to understand how other people feel*”), Adaptability EI (“*I try to see things as they really are without daydreaming or fantasizing about them*,” “*It is difficult for me to start new things*,” “*My way to overcome difficulties is to face one thing at a time*”), Stress Management EI (“*I believe I can be up to the most difficult situations*,” “*I know how to deal with unexpected problems*,” “*I can manage stress without becoming too nervous*”), and General Mood EI (“*It is difficult for me to enjoy life*,” “*I am a cheerful person*,” “*It is difficult for me to smile*”). The instrument had good reliability (α = 0.85) (Picconi et al., 2019).

*Measures of personality*. Personality traits were measured with the Big Five Questionnaire—Second Version (BFQ-2; Caprara et al., 2005). The BFQ-2 consisted of 134 items that measure the five personality traits that are: Extroversion (“*I seem to be an active and vigorous person*,” “*I am not a talkative person*,” “*I don’t usually converse with any traveling companions*”), Agreeableness (“*I believe that there is something good in every person*,” “*I always know how to meet other people’s needs*,” “*I rarely behave in unpleasant and rude manner*”), Conscientiousness (“*Before submitting a work, I spend a lot of time reviewing it*,” “*I don’t like to overthink things*”), Emotional Stability (“*I don’t often feel tense*,” “*I often feel agitated*”), and Openness to experience (“*Reading is one of my favorite activities*,” “*I don’t spend much time reading*”). The questionnaire includes a Lie scale to check the validity of subjective responses. All the data about personality traits were collected on a subsample of the original sample of 775 participants (women = 395; men = 380). Extroversion (α = 0.85), Agreeableness (α = 0.87), Conscientiousness (α = 0.85), Emotional Stability (α = 0.90), and Openness to experience (α = 0.79); test–retest reliability: Extroversion (*r* = 0.79), Agreeableness (*r* = 0.76), Conscientiousness (*r* = 0.77), Emotional Stability (*r* = . 83), and Openness to experience (*r* = 0.81) (Caprara et al., 2005).

*Measures of fluid intelligence.* Fluid intelligence was measured with Fluid Intelligence Test, which comprises 48 items (FIT; Romanelli and Saggino, 2014). The measure was developed though the Item Response Theory or IRT, a set of statistical models to evaluate latent variables (e.g., intelligence) based on responses on a given item. According to IRT, a greater level of the latent variable is associated with a higher probability of endorsing a particular item measuring the trait (Balsamo, 2017). All data on fluid intelligence were collected on a subsample of the original sample of 687 participants (women = 360; men = 327). The instrument had good reliability (KR-20 = 0.97). Specifically, the FIT measures the ability to analyze figures and understand the relationship between parts; the ability to manipulate and identify elements of abstract figures (Romanelli and Saggino, 2014).

*Procedure*. This is a non-pharmacological experimental study, in which subjects were recruited from the general population without mental and physical illnesses. Participants’ anonymity and privacy were guaranteed according to the Italian and European privacy laws (Italian law *n*. 196/2003 and EU GDPR 679/2016, respectively). Informed consent was obtained from all individual participants included in the study. The study was approved by the Department of Medicine and Aging Sciences, Italy (Protocol Number = 2,223/07.09.2021).

### 2.2. Statistical analyses

*Descriptive statistics and consistency of the EQ-i.* We computed the descriptive statistics (mean, standard deviations, skewness, and kurtosis) of each of the five dimensions of the EQ-i and the global score. We estimated the internal consistency with Cronbach’s Alphas.

*Relationships between personality traits, fluid intelligence, and EQ-i factors and total score*. To assess the association among fluid intelligence, personality traits, and EI, we estimated the correlations between the EQ-i factors and the total score, the big five-factors of personality, and the FIT. Correlations were calculated through Pearson’s coefficient.

Furthermore, Exploratory structural equation modeling (ESEM; Asparouhov and Muthén, 2009) was used. The estimation method was the maximum likelihood. In the basic model (M0), a common latent factor linked the observed variables, which were the five dimensions of the EQ-i; the five-factors of personality of BFQ-2; and the FIT total score. In M0, all loadings were set free. We compared three models in relation to the basic model (M0) in which loadings were set free:

Model 1, in which the loadings of the five dimensions of the EQ-I were set free, the loadings of the five-factors of personality were fixed to 0.30, while the loading of the FIT was fixed to 0.10.

Model 2, in which the loadings of the five dimensions of the EQ-I were set free, the loadings of the five-factors of personality were fixed to 0.10, while the loading of the FIT was fixed to 0.30.

Model 3, in which the loadings of the five dimensions of the EQ-I were set free, the loadings of the five-factors of personality were fixed to 0.50, while the loading of the FIT was fixed to 0.00 (Marsh et al., 2014).

Goodness-of-fit indices were the χ^2^, the ratio χ^2^/df, the root mean square error of approximation (RMSEA) and the corresponding confidence interval (90% RMSEA), the Comparative Fit Index (CFI), the Tucker–Lewis fit index (TLI), and the Akaike’s information criterion (AIC). Models with an acceptable fit should have χ^2^/*df* < 3, RMSEA < 0.08, and CFI and TLI > 0.90 (Hu and Bentler, 1999; Schermelleh-Engel et al., 2003). Akaike’s information criterion for comparing two or more models with smaller values represents a better fit of the hypothesized model (Hu and Bentler, 1999). Prior to model testing, Mardia’s normality test was used to assess the normality of the EQ-i by evaluating the kurtosis (Mardia’s normalized estimate = 0.465; Mardia, 1974). The low Mardia value confirmed the normality of the data.

*Influence of gender on the EI.* To assess whether the EI is influenced by gender, the differences in the mean between genders for each EQ-i subscale and the EQ-i total score were estimated with multiple *t*-tests according to the False Discovery Rate (FDR) procedure (Benjamini and Hochberg, 1995). For each *t*-test, the corresponding *p*-value is compared to the *p*-value estimated according to the FDR procedure (d*). If *p* is lower than d*, the difference is significant. Effect size (*Cohen’s d*) is also provided. The dummy coding for gender was 0 for women and 1 for men. Furthermore, we conducted a Multigroup Confirmatory Factor Analysis (MG-CFA) (Meredith, 1993) to assess the measurement invariance of the EQ-i with respect to the gender on a set of nested models assessed for the Italian standardization of the EQ-i (Franco and Tappatà, 2009):

1. The baseline configural invariance model (M1) in which the same factorial pattern was specified for each group, but with loadings and intercepts free to vary between occasions.

2. The metric invariance model (M2), wherein loadings were constrained to be equal across occasions.

3. The scalar invariance model (M3), wherein factor loadings and intercepts were constrained to be equal across conditions.

4. The strict invariance model wherein factor loadings, intercepts, and residual variances were constrained to be equal across occasions (M4).

Model fit was assessed using the χ^2^ statistical test, the Root mean Square Error of Approximation (RMSEA), and the Comparative Fit Index (CFI). The difference between CFIs (ΔCFI) of invariance models was estimated to assess measurement invariance. A value of ΔCFI smaller than or equal to | 0.01| (in absolute values) indicates that the null hypothesis of invariance should not be rejected (Cheung and Rensvold, 2002). Tests with scalar invariance are considered to be consistent tests, since they are not influenced by group characteristics (Meredith, 1993). In case of violation of total invariance of loadings or intercepts, partial invariance was also applied, leaving some parameters among groups free (Meredith, 1993). In the case of total or partial multigroup invariance with M3 or M4 models, we also assessed factor means difference across groups by setting up a model (M5) in which the factor means were zero in all groups. We estimated the significance of the difference between the chi-square value of M5 and that of M3 or M4. If the value of the difference was not significant, factor means could be considered equal among groups.

Descriptive statistics, EQ-i consistency, and correlations were performed using the SPSS 18.0.1 (SPSS Inc., Chicago, IL, USA) for Windows. The ESEM and the MG-CFA were analyzed using M-plus 7.0 (Muthén and Muthén, 1998–2012).

## 3. Results

Table 1 shows the descriptive statistics and internal consistency of the five dimensions and the global EQ-i scores in the total sample, the female sample, and the male sample.

**TABLE 1:** Descriptive statistics and the internal consistency of the five dimensions and the global score of the EQ-i in total, female, and male samples.

Samples	EQ-i subscales and total score	Mean	SD	Skewness	Kurtosis	Cronbach’s alpha
Total sample (*n* = 1,063)	Intrapersonal EI	140.09	16.67	-0.121	0.162	0.83
	Adaptability EI	84.53	10.15	0.406	0.521	0.70
	General mood EI	59.61	7.83	-0.106	0.466	0.69
	Interpersonal EI	108.15	12.04	-0.150	0.427	0.72
	Stress management EI	56.73	9.72	-0.054	-0.171	0.77
	EQ-i total score	449.12	43.77	0.198	0.551	0.91
Females (*n* = 578)	Intrapersonal EI	138.24	16.84	-0.150	0.150	0.83
	Adaptability EI	84.09	10.28	0.457	0.182	0.71
	General mood EI	59.16	8.11	-0.121	0.337	0.71
	Interpersonal EI	110.64	12.20	-0.376	0.963	0.73
	Stress management EI	55.24	9.63	-0.035	-0.373	0.77
	EQ-i total score	447.38	44.46	0.183	0.625	0.91
Males (*n* = 485)	Intrapersonal EI	142.30	16.20	-0.057	0.135	0.83
	Adaptability EI	85.05	9.97	0.353	1.025	0.70
	General mood EI	60.14	7.45	-0.044	0.615	0.67
	Interpersonal EI	105.19	11.15	0.019	0.159	0.68
	Stress management EI	58.51	9.53	-0.071	0.098	0.77
	EQ-i total score	451.20	42.89	0.230	0.458	0.92

Cronbach’s alphas were all acceptable (from 0.67 to 0.92) for each EQ-i dimension and the total score for the global, women, and men samples.

Table 2 shows correlations among the five dimensions and the global EQ-I score, personality traits, and the FIT. Results showed that the EQ-i factors, in general, had significant and positive correlations with the personality dimensions. The highest values were between the Interpersonal factor and the Agreeableness (*r* = 0.654; *p* < 0.01). Furthermore, in general, correlations showed no associations between EI and fluid intelligence. Moreover, results showed significant and positive correlations between the General Mood and the FIT (*r* = 0.110; *p* < 0.01).

**TABLE 2:** Correlations among the five dimensions and the global score of the EQ-i, the personality traits, and the FIT.

EQ-i subscales and total score	E	A	C	E	O	FIT
Intrapersonal EI	0.525**	0.340**	0.442**	0.390**	0.372**	0.071
Adaptability EI	0.299**	0.203**	0.285**	0.493**	0.374**	0.034
General mood EI	0.429**	0.388**	0.218**	0.242**	0.244**	0.110**
Interpersonal EI	0.130**	0.654**	0.381**	0.059	0.302**	0.014
Stress management EI	0.117**	0.288**	0.131**	0.760**	0.293**	0.082*
EQ-i total score	0.427**	0.516**	0.428**	0.525**	0.442**	0.082*

E, extroversion; A, agreeableness; C, conscientiousness; E, emotional stability; O, openness to experience; FIT, fluid intelligence test; p, significance; *p < 0.05 and **p < 0.01.

Table 3 shows the Goodness-of-fit indexes of the four tested models for ESEM analysis. Before performing the analysis, we performed an *a posteriori* power analysis for structural equation models (Moshagen and Erdfelder, 2016) to assess the adequacy of the sample size. With α = 0.05 and RMSEA = 0.08, the resulting power (1—β) was >0.99 for models with *df* = 32 and *df* = 38, respectively, confirming the adequacy of sample size. The best model was the M0 model, in which loadings were set free for all the EQ-I dimensions, the BFQ-2, and the FIT global score. The M3 model was the second model with better indexes, in which the loadings of the personality factors were set to 0.50 and the loading of the FIT was set to 0.10.

**TABLE 3:** The goodness-of-fit indexes of ESEM (*n* = 687).

Models	χ^2^	df	χ^2^/df	RMSEA	90% RMSEA	TLI	CFI	AIC
M0	163.388	32	5.11	0.077	0.066–0.089	0.925	0.956	18636.986
M1	642.927	38	16.92	0.121	0.110–0.131	0.816	0.873	18879.932
M2	818.824	38	21.55	0.173	0.163–0.183	0.623	0.739	19280.423
M3	260.322	38	6.85	0.092	0.082–0.103	0.893	0.926	18721.920

df, degrees of freedom; RMSEA, Root-Mean-Square Error of Approximation; TLI, Tucker-Lewis fit index; CFI, comparative fit index; AIC, Akaike’s information criterion. M0: one factor model with free loadings on all 11 observed variables (five EQi dimensions; five BFQ factors of personality and FIT global score); M1 one factor model with free loadings on EQi dimensions, loadings = 0.30 on BFQ factors and loading = 0.10 on FIT; M2 one factor model with free loadings on EQi dimensions, loadings = 0.10 on BFQ factors and loading = 0.30 on FIT; M3 one factor model with free loadings on EQi dimensions, loadings = 0.50 on BFQ factors and loading = 0.00 on FIT.

Table 4 shows that differences in means among genders were significant for the Intrapersonal, Stress Management, and Interpersonal EI factors, while no significant differences for the Adaptability and General Mood EI factors and the EQ-i total score were found. Table 1 showed that men outperformed women in the Intrapersonal and Stress Management EI factors, while women outperformed men in the Interpersonal EI factor.

**TABLE 4:** Multiple *t*-tests (two tails) with corresponding *p*-values for testing gender differences between the EQ-i dimensions and total EQ-i score.

EQ-i subscales and total score	*T*	*P*	d*	Cohen’s d
EQ-i total score	-1.419	0.156	0.050	0.087
Adaptability EI	-1.531	0.126	0.042	0.094
General mood EI	-2.039	0.042	0.033	0.126
Intrapersonal EI	-3.981	0.000	0.025	0.245
Stress management EI	-5.540	0.000	0.017	0.341
Interpersonal EI	7.536	0.000	0.008	0.464

d* are probabilities estimated with the FDR procedure (Benjamini and Hochberg, 1995). Effect sizes (Cohen’s d) are also provided. *P*-values lower than d* are significant.

Finally, Table 5 shows the results of the MG-CFA for the measurement invariance of the EQ-i.

**TABLE 5:** Goodness-of-fit indexes for measurement invariance of the EQ-i between men and women.

Models	χ^2^	df	RMSEA	90% RMSEA	TLI	CFI	SRMR	Model comparisons	| Δ CFI|
M1	10.869	4	0.057	0.017–0.099	0.983	0.997	0.014		
M2	12.420	8	0.032	0.000–0.065	0.998	0.995	0.025	M2–M1	**0.002**
M3	172.802	12	0.159	0.138–0.180	0.870	0.922	0.091	M3–M2	0.073
M3*	151.976	11	0.155	0.134–0.178	0.876	0.932	0.091	M3*–M2	0.063
M3**	42.491	10	0.078	0.055–0.103	0.968	0.984	0.037	M3**–M2	0.011
M3***	12.563	9	0.027	0.000–0.060	0.996	0.998	0.025	M3***–M2	**0.003**
M4	25.217	14	0.039	0.011–0.063	0.992	0.995	0.066	M4–M3***	**0.003**
M5	29.070	15	0.042	0.018–0.065	0.991	0.993	0.081		

ΔCFIs lower than | 0.01| are in bold type. M1: model for configural invariance; M2: model for metric invariance; M3: model for scalar invariance; M3*: model M3 with free intercept for the Intrapersonal EI factor; M3**: model M3 with free intercepts for the Intrapersonal and Interpersonal EI factors; M3***: model M3 with free intercepts for the Intrapersonal, Interpersonal and Stress Management EI factors; M4: model M3*** with fixed residual variances; M5: model M4 with latent factor means equal across groups. Bold values represent the ΔCFI values <0.01.

Metric invariance was confirmed, because the ΔCFI between M2 and M1 was smaller than | 0.01|. Scalar invariance was not confirmed, because the ΔCFI between M3 and M2 was greater than | 0.01|. Therefore, we assessed partial scalar invariance by letting free intercepts for some EQ-i dimensions. Partial scalar invariance for model M3 with free intercept for the Intrapersonal EI factor (model M3*) was not confirmed, because the ΔCFI between M3* and M2 is greater than | 0.01|. Partial scalar invariance for model M3 with free intercepts for the Intrapersonal and Interpersonal EI factors (model M3^**^) was not confirmed, because the ΔCFI between M3^**^ and M2 is greater than | 0.01|. Partial scalar invariance for model M3 with free intercepts for the Intrapersonal, Interpersonal, and Stress Management EI factors (model M3^***^) was confirmed, because the ΔCFI between M3^***^ and M2 is smaller than | 0.01|. Therefore, an effect of group characteristics on the intercepts of the Intrapersonal, Interpersonal, and Stress Management factors was found. Model M4 is model M3^***^ with fixed residual variances. Strict invariance was confirmed because the ΔCFI between M4 and M3^***^ is smaller than | 0.01|. The chi-square difference between M5 and M4 was equal to 3.853, higher than the critical value of χ^2^ = 3.841 for *df* = 1 and α = 0.05. The Wald test of the mean difference of the latent EI factor between women and men is *z* = 1.95, with *p* = 0.051. This result agrees with the non-significant *t*-test for differences in the global EQ-i score between genders reported in Table 4. Therefore, the means of the latent EI factor and the global EI score are equal between men and women.

## 4. Discussion

The first aim of our study was to analyze the association among the EQ-i, personality factors, and intelligence, particularly fluid intelligence, to clarify whether the EQ-i could be considered as a measure of trait EI or ability EI, or both. Therefore, in our study, we compared the global and subscale scores obtained on the EQ-i with measures obtained with the Big Five personality inventory, as a five-factor model of personality is the most used personality model in the scientific literature (Abood, 2019). For this reason, EI measured by EQ-i can be considered a trait EI (Di Fabio and Saklofske, 2018). In line with the literature, our results showed that the EI measured with EQ-i is related to the five personality factors (Bar-On, 1997; Dawda and Hart, 2000; Van der Zee et al., 2002; Franco and Tappatà, 2009; Van der Linden et al., 2012, 2017). Taking these data into account, we confirmed our first Hypothesis H1a according to which the general EI factor measured by the EQ-i is related to personality factors.

Furthermore, no data are available on the relationship between EQ-i scores and measures of intelligence. Therefore, we compared the EQ-i scores with a measure of fluid intelligence associated with emotional and behavioral regulation and abstraction abilities (Downey et al., 2014; Romanelli and Saggino, 2014; Li et al., 2020a,b). Trait EI showed a minimal connection with fluid intelligence, according to ESEM analysis, but this connection can be judged as negligible. Therefore, our result confirmed that EI, measured with EQ-I, has no significant connection with fluid intelligence, as previously shown by some studies (Bastian et al., 2005; Van der Linden et al., 2012). EI, measured by EQ-i, can be considered as a personality factor or a mixture of personality dimensions (Petrides, 2010; Van der Linden et al., 2010, 2012, 2016, 2017).

In relation to gender characteristics, our results showed that EQ-i scores for some subscales were affected by the gender characteristics of individuals. Men and women were equivalent for the Adaptability and General Mood EI factors. Men outperformed women in the Intrapersonal and Stress Management EI factors. In other words, men had higher levels of self-awareness, knowledge of their potential, ability to understand and express their emotions and thoughts, and ability to cope with stressful events or situations. These findings confirmed the results obtained by previous research (Reiff et al., 2001; De Weerdt and Rossi, 2012; Paskaran and Azman, 2020). Women outperformed men in the Interpersonal EI factor, the ability to understand others’ emotions, and this datum is confirmed by previous studies by Bar-On et al. (2000), Reiff et al. (2001), Alumran and Punamäki (2008), and De Weerdt and Rossi (2012).

Our results also confirmed the absence of gender differences in the EQ-i total score, in agreement with previous research (Palmer et al., 2003; Saklofske et al., 2007; Paskaran and Azman, 2020). The latent EI factor means between men and women were not significantly different, and measurement invariance analysis between genders showed that the factor structure and the factor loadings of the EQ-i subscales were quite similar between men and women. Differences were significant, especially in some subscale intercepts. Therefore, Hypothesis H2b, according to which gender only influences scores of specific EQ-I dimensions, was thus confirmed.

Our results are slightly different from those obtained in the Italian standardization of the EQ-I (Franco and Tappatà, 2009). The authors found that men had higher EI global scores than women. It must be said, however, that the authors did not perform an MG-CFA to assess measurement invariance. They simply performed several ANOVAs between scores obtained by men and women in each subscale and the global score. Since the global EI score was the sum of the raw scores of EI subscales, the effect of each subscale on the global score was not weighted with its loading on the general factor (Whitman et al., 2009; Tsaousis and Kazi, 2013). Therefore, it is likely that individual differences affected the global EI score more than necessary.

This article showed that gender characteristics have a constant effect on some of the EQ-i subscales. Thus, the effect of gender characteristics should be considered in interpreting the scores, especially at subscale levels. However, further research is needed to ascertain whether gender effects are constant for each subscale or may vary across nations with diverse cultures and social norms. If some cultures or societies impose different gender behavioral patterns, especially in social interactions, these patterns may probably affect gender differences in EQ-i scores.

A limit of our research is that the subjects are mainly young adults. Future research on gender differences in EI should also include older or elder people.

## 5. Conclusion

In conclusion, EQ-i is a reliable scale to measure trait EI. It has good internal consistency, and all EQ-i subscales are valid measures of the general factor of EI, as all loadings are higher than 0.30. However, gender characteristics affect scores in some subscales. Our results underlined that men can perceive, understand, and read their own emotions. They are capable to manage “*Life Events*,” the negative events of everyday life, and controlling the impulses before they become actions. Women can distinguish, recognize, and read others’ emotions. EI dimensions relate to individuals’ personality traits more than their cognitive abilities, and these connections are modulated by their gender characteristics. Therefore, the scoring of the EI dimensions should also consider the gender characteristics of the individuals. These results could be used for subsequent meta-analysis research on EI. Furthermore, our data could be used for protocols to enhance individual differences concerning emotional and personality factors in various contexts, such as an academic or clinical field.

### 5.1. Practical implications

Practical implications concern the applications of our results in a specific context. In the clinical context and the job performance field specific, psychotherapeutic programs may be used differently for men and women. For example, men might follow cognitive programs to manage negative irrational beliefs (Ellis and Grieger, 1986), whereas women might follow behavioral programs to manage their bodily signs of discomfort (Beck and Dozois, 2011). In psychological research, our results fit in the body of studies that analyze the psychometric properties of EI, to explore what the construct measures. We are in line with the hypothesis that EI is a mixture of personality dimensions (Petrides, 2010; Petrides et al., 2018). Finally, our results can be used in the academic field, through specific programs to increase the Interpersonal and Intrapersonal factors of EI that enable better academic performance.

## Data availability statement

The raw data supporting the conclusions of this article will be made available by the authors, without undue reservation.

## Ethics statement

The studies involving human participants were reviewed and approved by the Department of Medicine and Aging Sciences, University of Chieti, Italy. The patients/participants provided their written informed consent to participate in this study.

## Author contributions

MT assisted with the data analyses, collaborated in writing the manuscript, assisted in the design of the study, and collaborated in editing the final manuscript. MRS designed the study, recruited the sample, drafted the manuscript, and collaborated in editing the final manuscript. LP assisted with the design of the study, collaborated in the data analyses, and collaborated in writing the manuscript. AS assisted with the design of the study and collaborated in editing the final manuscript. All authors contributed to the article and approved the submitted version.
